# 1,1′-Bicyclo­propyl-1,1′-diyl 1,1′-biphenyl-2,2′-dicarboxyl­ate

**DOI:** 10.1107/S160053681201851X

**Published:** 2012-05-05

**Authors:** Hoong-Kun Fun, Ching Kheng Quah, Kai Xu

**Affiliations:** a X-ray Crystallography Unit, School of Physics, Universiti Sains Malaysia, 11800 USM, Penang, Malaysia; b School of Chemistry and Chemical Engineering, Nanjing University, Nanjing 210093, People’s Republic of China

## Abstract

In the title compound, C_20_H_16_O_4_, the two benzene rings form a dihedral angle of 45.70 (4)°. In the crystal, mol­ecules are linked *via* C—H⋯O inter­actions into layers lying parallel to the *bc* plane.

## Related literature

For the background to this study, see the first paper in this series: Fun, Quah, Wu & Zhang (2012 ▶). For related structures in this series, see: Fun, Lim, Quah & Wu (2012 ▶); Fun, Quah & Wu (2012 ▶). For standard bond-length data, see: Allen *et al.* (1987 ▶). For the preparation, see: Wu *et al.* (2012 ▶).
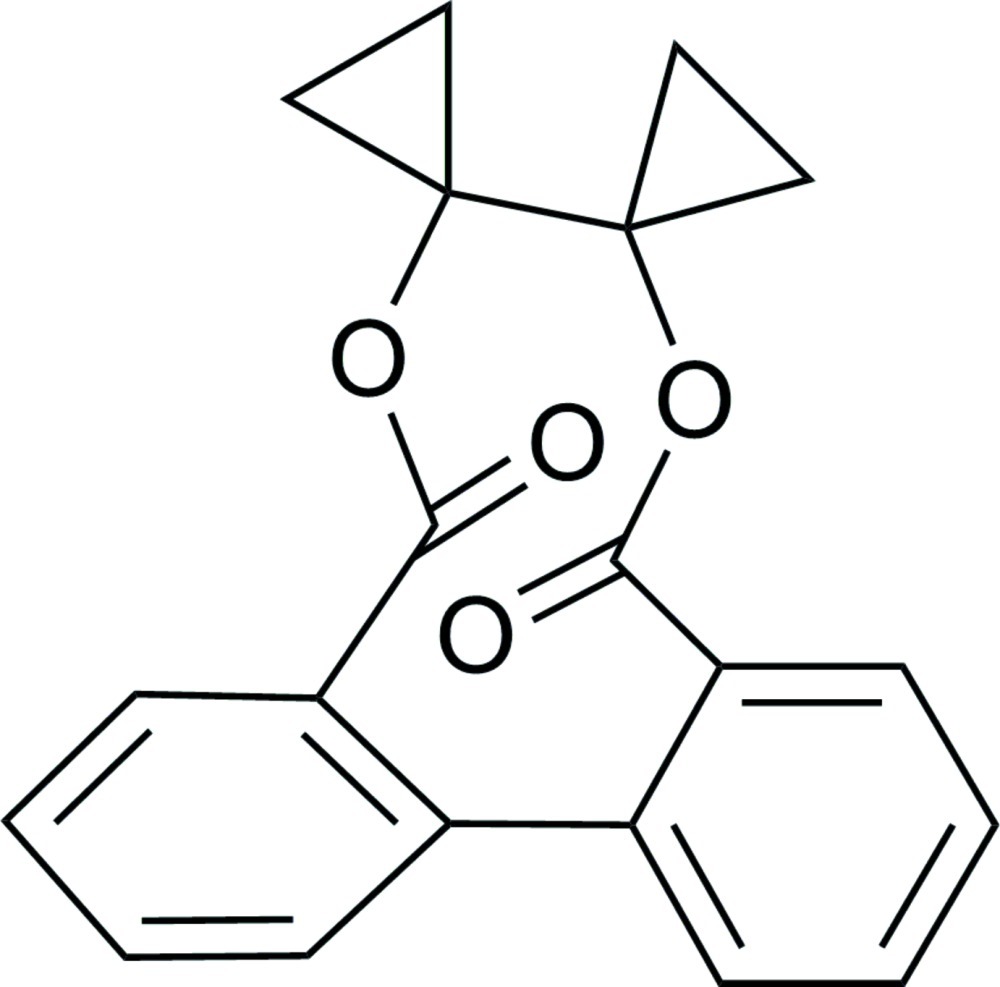


## Experimental

### 

#### Crystal data

C_20_H_16_O_4_
*M* *_r_* = 320.33
Monoclinic, 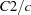

*a* = 26.3197 (14) Å
*b* = 9.4184 (5) Å
*c* = 13.3606 (7) Å
β = 100.092 (1)°
*V* = 3260.7 (3) Å^3^
*Z* = 8
Mo *K*α radiation
μ = 0.09 mm^−1^
*T* = 296 K
0.43 × 0.34 × 0.17 mm

#### Data collection

Bruker SMART APEXII DUO CCD area-detector diffractometer
Absorption correction: multi-scan (*SADABS*; Bruker, 2009 ▶) *T*_min_ = 0.962, *T*_max_ = 0.985
12233 measured reflections
5234 independent reflections
3687 reflections with *I* > 2σ(*I*)
*R*_int_ = 0.019

#### Refinement

*R*[*F* ^2^ > 2σ(*F* ^2^)] = 0.043
*wR*(*F* ^2^) = 0.124
*S* = 1.04
5234 reflections
217 parameters
H-atom parameters constrained
Δρ_max_ = 0.24 e Å^−3^
Δρ_min_ = −0.19 e Å^−3^

### 

Data collection: *APEX2* (Bruker, 2009 ▶); cell refinement: *SAINT* (Bruker, 2009 ▶); data reduction: *SAINT*; program(s) used to solve structure: *SHELXTL* (Sheldrick, 2008 ▶); program(s) used to refine structure: *SHELXTL*; molecular graphics: *SHELXTL*; software used to prepare material for publication: *SHELXTL* and *PLATON* (Spek, 2009 ▶).

## Supplementary Material

Crystal structure: contains datablock(s) global, I. DOI: 10.1107/S160053681201851X/is5125sup1.cif

Structure factors: contains datablock(s) I. DOI: 10.1107/S160053681201851X/is5125Isup2.hkl

Supplementary material file. DOI: 10.1107/S160053681201851X/is5125Isup3.cml

Additional supplementary materials:  crystallographic information; 3D view; checkCIF report

## Figures and Tables

**Table 1:** Hydrogen-bond geometry (Å, °)

*D*—H⋯*A*	*D*—H	H⋯*A*	*D*⋯*A*	*D*—H⋯*A*
C3—H3*A*⋯O3^i^	0.93	2.50	3.3410 (16)	151
C4—H4*A*⋯O3^ii^	0.93	2.52	3.4104 (15)	161
C10—H10*A*⋯O1^iii^	0.93	2.57	3.496 (2)	174

Symmetry codes: (i) 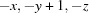; (ii) 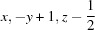; (iii) 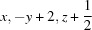.

**Fig. 1.**
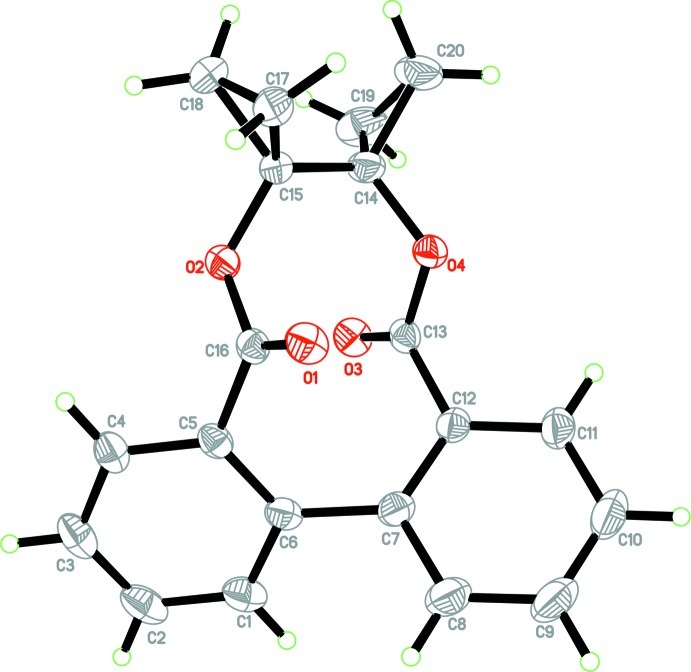
The molecular structure of the title compound, showing 30% probability displacement ellipsoids for non-H atoms.

**Fig. 2.**
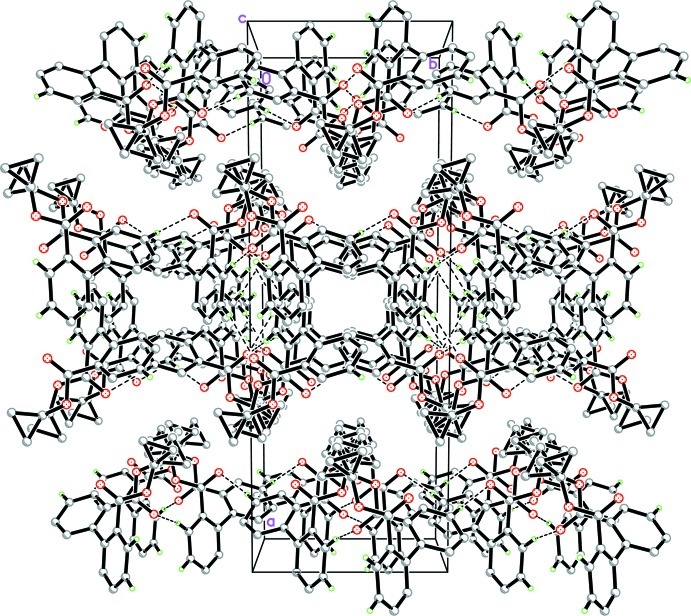
A packing diagram of the title compound, viewed along the c axis. H atoms not involved in hydrogen bonds (dashed lines) have been omitted for clarity.

## supplementary crystallographic information

### Comment

The title compound was firstly reported as a cyclopropyl ring-fused ten-membered bislactone whose preparation could be achieved through a concise photochemical method (Wu *et al.*, 2012). In the title compound, Fig. 1, the benzene rings (C1–C6 and C7–C12) form a dihedral angle of 45.70 (4)°. Bond lengths (Allen *et al.*, 1987) and angles are within normal ranges and are comparable to related structures (Fun, Quah, Wu & Zhang, 2012; Fun, Lim, Quah & Wu, 2012; Fun, Quah & Wu, 2012). In the crystal (Fig. 2), molecules are linked *via* intermolecular C3—H3A···O3, C4—H4A···O3 and C10—H10A···O1 interactions (Table 1) into a layer parallel to the (100) plane.

### Experimental

The title compound was derived from the photo-induced sequential reactions of 9,10-phenanthrenedione with bicyclopropylendiene. The compound was purified by flash column chromatography with ethyl acetate/petroleum ether (1:10) as eluents. X-ray quality crystals of the title compound (*m.p.* 192–194 °C) were obtained from slow evaporation of an acetone and petroleum ether solution (1:10).

### Refinement

All H atoms were positioned geometrically and refined using a riding model with C—H = 0.93 or 0.97 Å and *U*_iso_(H) = 1.2*U*_eq_(C).

### Crystal data

**Table:**

C_20_H_16_O_4_	*F*(000) = 1344
*M**_r_* = 320.33	*D*_x_ = 1.305 Mg m^−^^3^
Monoclinic, *C*2/*c*	Mo *K*α radiation, λ = 0.71073 Å
Hall symbol: -C 2yc	Cell parameters from 5289 reflections
*a* = 26.3197 (14) Å	θ = 2.7–31.1°
*b* = 9.4184 (5) Å	µ = 0.09 mm^−^^1^
*c* = 13.3606 (7) Å	*T* = 296 K
β = 100.092 (1)°	Block, colourless
*V* = 3260.7 (3) Å^3^	0.43 × 0.34 × 0.17 mm
*Z* = 8	

### Data collection

**Table:**

Bruker SMART APEXII DUO CCD area-detector diffractometer	5234 independent reflections
Radiation source: fine-focus sealed tube	3687 reflections with *I* > 2σ(*I*)
Graphite monochromator	*R*_int_ = 0.019
φ and ω scans	θ_max_ = 31.2°, θ_min_ = 2.3°
Absorption correction: multi-scan (*SADABS*; Bruker, 2009)	*h* = −38→30
*T*_min_ = 0.962, *T*_max_ = 0.985	*k* = −10→13
12233 measured reflections	*l* = −19→14

### Refinement

**Table:**

Refinement on *F*^2^	Primary atom site location: structure-invariant direct methods
Least-squares matrix: full	Secondary atom site location: difference Fourier map
*R*[*F*^2^ > 2σ(*F*^2^)] = 0.043	Hydrogen site location: inferred from neighbouring sites
*wR*(*F*^2^) = 0.124	H-atom parameters constrained
*S* = 1.04	*w* = 1/[σ^2^(*F*_o_^2^) + (0.0571*P*)^2^ + 0.6692*P*] where *P* = (*F*_o_^2^ + 2*F*_c_^2^)/3
5234 reflections	(Δ/σ)_max_ = 0.001
217 parameters	Δρ_max_ = 0.24 e Å^−^^3^
0 restraints	Δρ_min_ = −0.18 e Å^−^^3^

### Special details

**Table:**

Geometry. All esds (except the esd in the dihedral angle between two l.s. planes) are estimated using the full covariance matrix. The cell esds are taken into account individually in the estimation of esds in distances, angles and torsion angles; correlations between esds in cell parameters are only used when they are defined by crystal symmetry. An approximate (isotropic) treatment of cell esds is used for estimating esds involving l.s. planes.
Refinement. Refinement of F^2^ against ALL reflections. The weighted R-factor wR and goodness of fit S are based on F^2^, conventional R-factors R are based on F, with F set to zero for negative F^2^. The threshold expression of F^2^ > 2sigma(F^2^) is used only for calculating R-factors(gt) etc. and is not relevant to the choice of reflections for refinement. R-factors based on F^2^ are statistically about twice as large as those based on F, and R- factors based on ALL data will be even larger.

### Fractional atomic coordinates and isotropic or equivalent isotropic displacement parameters (Å^2^)

**Table:**

	*x*	*y*	*z*	*U*_iso_*/*U*_eq_	
O1	0.16306 (3)	0.77985 (9)	0.06344 (6)	0.0449 (2)	
O2	0.14795 (3)	0.54239 (8)	0.06055 (6)	0.03836 (18)	
O3	0.09630 (3)	0.53439 (9)	0.24353 (7)	0.0472 (2)	
O4	0.17759 (3)	0.62505 (9)	0.26443 (6)	0.03936 (19)	
C1	−0.00465 (5)	0.77534 (15)	0.06104 (11)	0.0523 (3)	
H1A	−0.0238	0.8243	0.1021	0.063*	
C2	−0.02987 (5)	0.70956 (16)	−0.02684 (12)	0.0591 (4)	
H2A	−0.0656	0.7157	−0.0442	0.071*	
C3	−0.00266 (5)	0.63585 (15)	−0.08813 (10)	0.0549 (4)	
H3A	−0.0197	0.5930	−0.1473	0.066*	
C4	0.05052 (5)	0.62568 (13)	−0.06123 (9)	0.0447 (3)	
H4A	0.0691	0.5737	−0.1017	0.054*	
C5	0.07625 (4)	0.69263 (11)	0.02585 (8)	0.0354 (2)	
C6	0.04899 (4)	0.76921 (12)	0.08875 (9)	0.0390 (2)	
C7	0.07330 (4)	0.84548 (12)	0.18338 (9)	0.0412 (3)	
C8	0.05919 (5)	0.98669 (15)	0.19508 (13)	0.0598 (4)	
H8A	0.0359	1.0299	0.1437	0.072*	
C9	0.07889 (6)	1.06313 (17)	0.28054 (15)	0.0731 (5)	
H9A	0.0687	1.1568	0.2864	0.088*	
C10	0.11352 (6)	1.00248 (18)	0.35741 (14)	0.0687 (5)	
H10A	0.1263	1.0542	0.4157	0.082*	
C11	0.12910 (5)	0.86488 (16)	0.34756 (10)	0.0521 (3)	
H11A	0.1533	0.8245	0.3987	0.063*	
C12	0.10904 (4)	0.78468 (13)	0.26178 (9)	0.0387 (2)	
C13	0.12509 (4)	0.63393 (12)	0.25576 (7)	0.0357 (2)	
C14	0.19823 (4)	0.49827 (13)	0.22704 (9)	0.0429 (3)	
C15	0.19826 (4)	0.50931 (12)	0.11551 (8)	0.0384 (2)	
C16	0.13365 (4)	0.68165 (12)	0.05129 (7)	0.0343 (2)	
C17	0.24439 (5)	0.55872 (15)	0.07486 (10)	0.0489 (3)	
H17A	0.2754	0.5802	0.1233	0.059*	
H17B	0.2387	0.6182	0.0147	0.059*	
C18	0.22764 (5)	0.40633 (15)	0.06318 (11)	0.0538 (3)	
H18A	0.2119	0.3740	−0.0040	0.065*	
H18B	0.2485	0.3360	0.1046	0.065*	
C19	0.19195 (7)	0.36209 (18)	0.27970 (13)	0.0696 (4)	
H19A	0.1885	0.2759	0.2393	0.084*	
H19B	0.1722	0.3634	0.3346	0.084*	
C20	0.24300 (6)	0.4358 (2)	0.29662 (11)	0.0694 (5)	
H20A	0.2542	0.4817	0.3617	0.083*	
H20B	0.2705	0.3943	0.2665	0.083*	

### Atomic displacement parameters (Å^2^)

**Table:**

	*U*^11^	*U*^22^	*U*^33^	*U*^12^	*U*^13^	*U*^23^
O1	0.0411 (4)	0.0398 (5)	0.0524 (5)	−0.0053 (3)	0.0041 (3)	0.0041 (4)
O2	0.0387 (4)	0.0360 (4)	0.0382 (4)	0.0023 (3)	0.0007 (3)	−0.0012 (3)
O3	0.0456 (4)	0.0456 (5)	0.0504 (5)	−0.0062 (4)	0.0084 (4)	0.0037 (4)
O4	0.0337 (4)	0.0490 (5)	0.0351 (4)	0.0054 (3)	0.0052 (3)	−0.0027 (3)
C1	0.0362 (6)	0.0546 (8)	0.0642 (8)	0.0055 (5)	0.0043 (5)	0.0055 (6)
C2	0.0369 (6)	0.0623 (9)	0.0709 (9)	−0.0037 (6)	−0.0102 (6)	0.0134 (7)
C3	0.0519 (7)	0.0561 (8)	0.0486 (7)	−0.0126 (6)	−0.0135 (6)	0.0087 (6)
C4	0.0500 (6)	0.0437 (7)	0.0370 (5)	−0.0049 (5)	−0.0017 (5)	0.0032 (5)
C5	0.0360 (5)	0.0331 (5)	0.0351 (5)	−0.0022 (4)	0.0005 (4)	0.0064 (4)
C6	0.0351 (5)	0.0359 (6)	0.0442 (6)	0.0029 (4)	0.0020 (4)	0.0045 (5)
C7	0.0367 (5)	0.0373 (6)	0.0502 (6)	0.0015 (4)	0.0091 (5)	−0.0051 (5)
C8	0.0554 (7)	0.0439 (7)	0.0795 (10)	0.0075 (6)	0.0101 (7)	−0.0091 (7)
C9	0.0669 (9)	0.0481 (8)	0.1057 (13)	0.0041 (7)	0.0188 (9)	−0.0300 (9)
C10	0.0543 (8)	0.0691 (10)	0.0843 (11)	−0.0093 (7)	0.0165 (7)	−0.0430 (9)
C11	0.0368 (6)	0.0655 (9)	0.0539 (7)	−0.0037 (5)	0.0077 (5)	−0.0234 (6)
C12	0.0305 (5)	0.0437 (6)	0.0430 (6)	−0.0016 (4)	0.0099 (4)	−0.0089 (5)
C13	0.0352 (5)	0.0447 (6)	0.0273 (4)	0.0002 (4)	0.0059 (4)	0.0003 (4)
C14	0.0445 (6)	0.0479 (7)	0.0364 (5)	0.0152 (5)	0.0072 (4)	0.0055 (5)
C15	0.0379 (5)	0.0409 (6)	0.0353 (5)	0.0081 (4)	0.0036 (4)	−0.0031 (4)
C16	0.0380 (5)	0.0364 (6)	0.0280 (4)	0.0007 (4)	0.0046 (4)	0.0027 (4)
C17	0.0420 (6)	0.0606 (8)	0.0451 (6)	0.0037 (5)	0.0105 (5)	−0.0069 (6)
C18	0.0544 (7)	0.0559 (8)	0.0505 (7)	0.0153 (6)	0.0079 (5)	−0.0132 (6)
C19	0.0852 (11)	0.0595 (9)	0.0680 (10)	0.0263 (8)	0.0240 (8)	0.0266 (8)
C20	0.0645 (9)	0.0966 (12)	0.0456 (7)	0.0407 (9)	0.0053 (6)	0.0153 (8)

### Geometric parameters (Å, º)

**Table:**

O1—C16	1.1986 (13)	C9—C10	1.373 (2)
O2—C16	1.3641 (13)	C9—H9A	0.9300
O2—C15	1.4314 (12)	C10—C11	1.373 (2)
O3—C13	1.1984 (13)	C10—H10A	0.9300
O4—C13	1.3688 (12)	C11—C12	1.3967 (16)
O4—C14	1.4372 (14)	C11—H11A	0.9300
C1—C2	1.389 (2)	C12—C13	1.4875 (16)
C1—C6	1.3966 (15)	C14—C19	1.486 (2)
C1—H1A	0.9300	C14—C20	1.4883 (16)
C2—C3	1.368 (2)	C14—C15	1.4939 (16)
C2—H2A	0.9300	C15—C18	1.4889 (17)
C3—C4	1.3859 (17)	C15—C17	1.4889 (17)
C3—H3A	0.9300	C17—C18	1.502 (2)
C4—C5	1.3906 (15)	C17—H17A	0.9700
C4—H4A	0.9300	C17—H17B	0.9700
C5—C6	1.3972 (17)	C18—H18A	0.9700
C5—C16	1.4930 (14)	C18—H18B	0.9700
C6—C7	1.4967 (16)	C19—C20	1.494 (3)
C7—C8	1.3970 (18)	C19—H19A	0.9700
C7—C12	1.4017 (16)	C19—H19B	0.9700
C8—C9	1.372 (2)	C20—H20A	0.9700
C8—H8A	0.9300	C20—H20B	0.9700
			
C16—O2—C15	118.13 (8)	O4—C13—C12	110.23 (9)
C13—O4—C14	117.31 (9)	O4—C14—C19	118.18 (11)
C2—C1—C6	121.11 (13)	O4—C14—C20	114.67 (11)
C2—C1—H1A	119.4	C19—C14—C20	60.30 (11)
C6—C1—H1A	119.4	O4—C14—C15	110.81 (9)
C3—C2—C1	120.63 (12)	C19—C14—C15	123.50 (12)
C3—C2—H2A	119.7	C20—C14—C15	120.79 (11)
C1—C2—H2A	119.7	O2—C15—C18	114.25 (9)
C2—C3—C4	119.39 (12)	O2—C15—C17	119.02 (10)
C2—C3—H3A	120.3	C18—C15—C17	60.56 (9)
C4—C3—H3A	120.3	O2—C15—C14	111.39 (9)
C3—C4—C5	120.48 (13)	C18—C15—C14	120.94 (10)
C3—C4—H4A	119.8	C17—C15—C14	121.91 (10)
C5—C4—H4A	119.8	O1—C16—O2	124.63 (10)
C4—C5—C6	120.75 (10)	O1—C16—C5	125.52 (10)
C4—C5—C16	118.95 (11)	O2—C16—C5	109.84 (9)
C6—C5—C16	120.30 (9)	C15—C17—C18	59.72 (9)
C1—C6—C5	117.62 (11)	C15—C17—H17A	117.8
C1—C6—C7	117.82 (11)	C18—C17—H17A	117.8
C5—C6—C7	124.57 (9)	C15—C17—H17B	117.8
C8—C7—C12	117.51 (11)	C18—C17—H17B	117.8
C8—C7—C6	117.96 (11)	H17A—C17—H17B	114.9
C12—C7—C6	124.52 (10)	C15—C18—C17	59.72 (8)
C9—C8—C7	121.57 (14)	C15—C18—H18A	117.8
C9—C8—H8A	119.2	C17—C18—H18A	117.8
C7—C8—H8A	119.2	C15—C18—H18B	117.8
C8—C9—C10	120.58 (14)	C17—C18—H18B	117.8
C8—C9—H9A	119.7	H18A—C18—H18B	114.9
C10—C9—H9A	119.7	C14—C19—C20	59.92 (10)
C11—C10—C9	119.44 (13)	C14—C19—H19A	117.8
C11—C10—H10A	120.3	C20—C19—H19A	117.8
C9—C10—H10A	120.3	C14—C19—H19B	117.8
C10—C11—C12	120.87 (13)	C20—C19—H19B	117.8
C10—C11—H11A	119.6	H19A—C19—H19B	114.9
C12—C11—H11A	119.6	C14—C20—C19	59.78 (9)
C11—C12—C7	120.00 (11)	C14—C20—H20A	117.8
C11—C12—C13	119.38 (11)	C19—C20—H20A	117.8
C7—C12—C13	120.61 (10)	C14—C20—H20B	117.8
O3—C13—O4	124.64 (11)	C19—C20—H20B	117.8
O3—C13—C12	125.13 (10)	H20A—C20—H20B	114.9
			
C6—C1—C2—C3	0.6 (2)	C7—C12—C13—O4	123.65 (11)
C1—C2—C3—C4	0.8 (2)	C13—O4—C14—C19	−66.02 (14)
C2—C3—C4—C5	−1.62 (19)	C13—O4—C14—C20	−134.16 (12)
C3—C4—C5—C6	1.13 (17)	C13—O4—C14—C15	84.84 (12)
C3—C4—C5—C16	−178.81 (11)	C16—O2—C15—C18	−132.94 (11)
C2—C1—C6—C5	−1.04 (19)	C16—O2—C15—C17	−64.44 (13)
C2—C1—C6—C7	178.72 (12)	C16—O2—C15—C14	85.60 (12)
C4—C5—C6—C1	0.20 (17)	O4—C14—C15—O2	−54.61 (13)
C16—C5—C6—C1	−179.86 (11)	C19—C14—C15—O2	94.42 (14)
C4—C5—C6—C7	−179.54 (11)	C20—C14—C15—O2	167.13 (12)
C16—C5—C6—C7	0.40 (17)	O4—C14—C15—C18	166.88 (11)
C1—C6—C7—C8	−49.77 (17)	C19—C14—C15—C18	−44.10 (18)
C5—C6—C7—C8	129.97 (13)	C20—C14—C15—C18	28.61 (19)
C1—C6—C7—C12	129.69 (13)	O4—C14—C15—C17	94.44 (13)
C5—C6—C7—C12	−50.56 (18)	C19—C14—C15—C17	−116.54 (15)
C12—C7—C8—C9	−0.9 (2)	C20—C14—C15—C17	−43.82 (18)
C6—C7—C8—C9	178.62 (14)	C15—O2—C16—O1	18.83 (16)
C7—C8—C9—C10	0.3 (3)	C15—O2—C16—C5	−160.13 (9)
C8—C9—C10—C11	1.1 (3)	C4—C5—C16—O1	123.22 (13)
C9—C10—C11—C12	−1.9 (2)	C6—C5—C16—O1	−56.72 (16)
C10—C11—C12—C7	1.3 (2)	C4—C5—C16—O2	−57.83 (13)
C10—C11—C12—C13	−177.34 (13)	C6—C5—C16—O2	122.23 (11)
C8—C7—C12—C11	0.07 (18)	O2—C15—C17—C18	−103.08 (11)
C6—C7—C12—C11	−179.39 (11)	C14—C15—C17—C18	110.12 (12)
C8—C7—C12—C13	178.70 (12)	O2—C15—C18—C17	110.90 (11)
C6—C7—C12—C13	−0.76 (18)	C14—C15—C18—C17	−111.67 (13)
C14—O4—C13—O3	19.37 (15)	O4—C14—C19—C20	−103.86 (13)
C14—O4—C13—C12	−159.59 (9)	C15—C14—C19—C20	109.22 (14)
C11—C12—C13—O3	123.34 (13)	O4—C14—C20—C19	109.65 (13)
C7—C12—C13—O3	−55.30 (16)	C15—C14—C20—C19	−113.56 (15)
C11—C12—C13—O4	−57.71 (14)		

### Hydrogen-bond geometry (Å, º)

**Table:**

*D*—H···*A*	*D*—H	H···*A*	*D*···*A*	*D*—H···*A*
C3—H3*A*···O3^i^	0.93	2.50	3.3410 (16)	151
C4—H4*A*···O3^ii^	0.93	2.52	3.4104 (15)	161
C10—H10*A*···O1^iii^	0.93	2.57	3.496 (2)	174

Symmetry codes: (i) −*x*, −*y*+1, −*z*; (ii) *x*, −*y*+1, *z*−1/2; (iii) *x*, −*y*+2, *z*+1/2.
